# Migraine and risk of adverse obstetric outcomes: a matched cohort study of English linked electronic health records

**DOI:** 10.1186/s12916-026-04991-w

**Published:** 2026-07-07

**Authors:** Katherine Phillips, Megha Singh, Siang Ing Lee, Nadia Sarween, Kelly-Ann Eastwood, Arturo Gonzalez-Izquierdo, Benjamin Wakerley, Lakshrieni Nishshanka, Neil Cockburn, Francesca L Crowe, Krishnarajah Nirantharakumar

**Affiliations:** 1 Department of Applied Health Sciences, College of Medicine and Health, University of Birmingham, Edgbaston, Birmingham, B15 2TT UK; 2 Centre for Public Health, Queen’s University of Belfast, BT7 1NN Belfast, UK; 3 St Michael’s Hospital, University Hospitals Bristol NHS Foundation Trust, BS2 8EG Bristol , UK; 4 Department of Metabolism and Systems Science, University of Birmingham, Birmingham, B15 2TT UK; 5 Department of Applied Health, University of Warwick, Coventry, UK; 6 School of Life Course & Population Sciences, King’s College London, London, UK

**Keywords:** Migraine, preterm birth, pregnancy complications

## Abstract

**Background:**

Existing literature suggests a link between migraine and various adverse pregnancy outcomes, but findings remain inconsistent. This study aimed to evaluate the association between pre-pregnancy migraine and obstetric outcomes.

**Methods:**

This retrospective cohort study used linked electronic health records from Clinical Practice Research Datalink (CPRD) GOLD and Hospital Episode Statistics (HES) maternity data. We included women aged 15–50 years in England with singleton deliveries between 2000 and 2019. Deliveries from women with a recorded migraine diagnosis before pregnancy were matched 1:1 by maternal age to those without migraine. Logistic regression models, adjusted for demographic and clinical factors, were used to examine associations with preterm birth, low birth weight, small for gestational age (SGA), mode of delivery, and stillbirth.

**Results:**

The cohort included 428,217 deliveries from 317,016 women, comprising 46,560 (10.9%) migraine-exposed deliveries and an equal number of matched controls. No significant association was found between pre-pregnancy migraine and preterm birth (aOR 1.04(0.99–1.09)), although migraine appeared to have a protective effect against very preterm birth (aOR 0.83(0.73–0.95)). Pre-pregnancy migraine was associated with increased odds of iatrogenic preterm birth (aOR 1.10, 95% CI: 1.04–1.18), but not spontaneous preterm birth (aOR 0.97, 95% CI: 0.91–1.03). Migraine was associated with a significant increase in the odds of pre-eclampsia (aOR 1.17 (95% CI 1.07–1.27)). There were no notable associations with low birth weight (1500–2500 g) (aOR 1.02, 95% CI: 0.95–1.09), very low (1000–1500 g) or extremely low birth weight (< 1000 g), SGA (aOR 0.96, 95% CI: 0.89–1.03), or stillbirth (aOR 1.00, 95% CI: 0.81–1.24). No significant association was seen between migraine and elective caesarean section (aOR 1.04, 95% CI: 0.99–1.09).

**Conclusion:**

Pre-pregnancy migraine was associated with increased odds of iatrogenic preterm birth and pre-eclampsia. Associations with other outcomes were small and uncertain. Further research is needed to understand the mechanisms underlying these findings and their clinical relevance.

**Supplementary Information:**

The online version contains supplementary material available at https://doi.org/10.1186/s12916-026-04991-w.

## Background

Migraine is a common neurovascular disorder, affecting approximately one in seven people globally and disproportionately impacting women of reproductive age [1–3]. Nearly 30% of women experience migraine by age 45, and it is a major contributor to global disability, particularly among women aged 15–49 [4]. Though often considered benign, migraine is associated with significant morbidity, including an increased risk of cardiovascular disease and stroke, especially in women with migraine with aura [5–11]. These vascular implications may extend to pregnancy, with reported associations between migraine and outcomes such as pre-eclampsia, miscarriage, preterm birth, low birth weight, and small for gestational age (SGA) infants [12].

Several studies have examined migraine in relation to adverse pregnancy outcomes, but findings remain inconsistent. A 2019 meta-analysis reported associations with pre-eclampsia and low birth weight while a Danish cohort study found increased risks of miscarriage, preterm birth, pregnancy-induced hypertension, and caesarean delivery [13, 14]. Biological mechanisms proposed include systemic inflammation, endothelial dysfunction, and hypercoagulability processes implicated in pre-eclampsia and preterm birth [5–10]. Migraine treatments during pregnancy, such as paracetamol, Non-steroidal anti-inflammatory drugs (NSAIDs), and beta-blockers, have limited safety data, and early NSAID use may increase miscarriage risk [15–18]. Despite a growing body of research, uncertainties remain regarding the extent and mechanisms by which migraine may influence obstetric outcomes.

Key gaps in the literature include limited differentiation between spontaneous and iatrogenic preterm birth, despite their distinct causes and implications [19–21]. A recent meta-analysis reported a pooled adjusted odds ratio (aOR) of 1.32 (95% CI: 1.15–1.51) for preterm birth among women with migraine [12], driven largely by three cohort studies that did not distinguish between preterm birth subtypes [22–24].

Evidence for low birth weight and SGA is also mixed. One study found an association between migraine and SGA (aOR 1.08, 95% CI: 1.01–1.15) but not with low birth weight after adjustment [13]. A systematic review similarly found associations with SGA but not with low birth weight in studies reporting adjusted analyses [12, 22, 25]. Migraine has also been linked to a 16–20% increased risk of caesarean delivery procedures [23, 29, 30] which have different clinical indications and implications [26, 27] that warrant further investigation. However, studies have not distinguished between elective and emergency caesarean delivery. No consistent association has been reported between migraine and stillbirth.

The aim of this study is to investigate the relationship between migraine and preterm birth (including spontaneous versus iatrogenic), low birth weight, small for gestational age, pre-eclampsia, mode of delivery (including emergency versus elective) and stillbirth.

## Methods

### Study design and data source

A retrospective cohort study of pregnant women with migraine matched to pregnant women without migraine was conducted to assess the association of pre-pregnancy migraine with obstetric outcomes. Data from this study derived from Clinical Practice Research Datalink (CPRD) GOLD linked to Hospital Episode Statistics (HES) maternity tail. CPRD GOLD is a dataset of electronic health records (EHRs) from General Practices (GPs) using Vision software. It contains data on patient demographics, diagnoses, symptoms and prescriptions and covers 7% of the UK population across almost 1000 practices [28]. The HES Admitted Patient Care dataset comprises data on all admissions to National Health Service (NHS) hospitals. When a birth admission occurs in an NHS hospital, delivery episodes are generated in the mother’s records. This includes a maternity tail which has information about birth outcomes including method of onset of labour, live and still birth, length of gestation and birth weight (29). HES linkage with CPRD GOLD is available for patients in England.

### Study population

Women aged 15–50 years with a singleton delivery recorded in HES maternity tail between January 1, 2000, and December 31, 2019, were eligible for inclusion. The study population was selected from patients within the CPRD primary care dataset who met the following data quality requirements: (1) having an acceptable patient flag within CPRD GOLD (which indicates sufficient data quality) (2) prior to pregnancy, a minimum of 1 year of registration with a practice or 1 year of registration after the up-to-standard date of the registered practice, whichever was later. Deliveries formed the unit of analysis and only singleton deliveries were included.

### Exposure, control and outcome definitions

Migraine, the exposure, was defined by the presence of a Read code indicating a clinical diagnosis or a single prescription of medications used exclusively in the management of migraine in UK practice (triptans, migraine combination treatments (acetylsalicylic acid or paracetamol, combined with codeine, caffeine and/or an antiemetic) or calcitonin gene-related peptide (CGRP) inhibitors) at any time prior to the pregnancy start date. Exact matching on maternal age (± 1 year) at the start of pregnancy and year of pregnancy start was used to match (1:1) deliveries of women with migraine to deliveries of women without migraine. Women with a history of any headache were excluded from the unexposed population as it was anticipated that there would be a number of women with a history of migraine who did not have it recorded.

Preterm birth was defined as a gestational age less than 37 weeks as recorded in the maternity tail or International Classification of Disease (ICD) 10 codes for preterm birth in the secondary care records. Where gestational age was recorded, preterm birth was further sub-divided into very preterm (28–32 weeks) and extremely preterm (< 28 weeks). Where onset of delivery was recorded in the maternity tail, preterm birth was sub-divided into spontaneous or iatrogenic (where onset was by caesarean or medical or surgical induction).

Low birth weight was defined as having a birth weight of less than 2500 g recorded in the HES maternity tail. This was sub-divided into low birth weight (LBW) (1500–2500 g) very low birth weight (VLBW) (1000–1500 g) and extremely low birth weight (ELBW) (< 1000 g). Birth weight was recorded in the maternity tail, but no ICD10 codes were available for low birth weight within the secondary care records.

Small for gestational age was defined as birth weight below the 10th centile for gestational age as compared to international standards. Birth weight, gestational age and fetal gender were entered into INTER-GROWTH [30] to calculate the weight for gestational age centile.

Pre-eclampsia was defined by a Read code or ICD10 indicating diagnosis in the primary or secondary care record respectively.

Mode of delivery was classified using the delivery method recorded in the maternity tail, ICD10 and Office of Population Censuses and Surveys Classification of Interventions and Procedures (OPCS) codes into the following categories: vaginal delivery, emergency caesarean, elective caesarean and unknown.

Stillbirth (birth of an infant that has died in the womb (strictly, after having survived through at least the first 24 weeks of pregnancy) was defined by the birth status variable in the maternity tail.

### Statistical methods

Data related to obstetric outcomes and secondary care diagnoses and procedures were obtained from HES Admitted Patient Care provided by CPRD. Extraction of primary care recorded demographic, lifestyle, diagnostic and prescription variables was facilitated using the data extraction for epidemiological research (DExtER) tool [31].

Deliveries were the unit of analysis for this study. Summary statistics for baseline characteristics were stratified by exposure to migraine prior to pregnancy. Frequencies and percentages were reported for binary variables. Median and interquartile range were reported for non-normally distributed continuous variables.

Logistic regression was performed for binary outcomes (preterm, low birth weight, SGA, stillbirth) and multinomial logistic regression was performed for categorical outcomes (mode of delivery and type of preterm) to provide unadjusted and adjusted odds ratios. Covariates were selected based on what previous similar studies had adjusted for [22, 23, 32, 33] and from a literature search for conditions associated with both migraine and adverse obstetric outcomes.

Models were adjusted for age bands (15–19, 20–24, 25–29, 30–34, 35–39, 40–44, 45–50 years), year of pregnancy, ethnicity (White, Asian, Black, other and missing), Index of Multiple Deprivation (IMD) in quintiles (1 – most deprived, 2, 3, 4, 5 – least deprived and missing), latest Body Mass Index (BMI) prior to conception (underweight (< 18.5), normal weight (18.5–24.9), overweight (25-<30), obese (≥ 30) kg/m^2^ and missing), pre-conception smoking status (non-smoker, ex-smoker, smoker and missing), pre-existing diabetes, asthma, systemic lupus erythematous, thyroid disorders, hypertension, chronic kidney disease, gestational diabetes and pre-eclampsia. As a woman could have more than one delivery during the study period, statistical inference from the log binomial regression model was based on cluster-robust standard errors to account for similarity of outcomes within a woman.

For the primary analysis where outcome data is missing, it was assumed that no adverse event occurs (or vaginal delivery in the case of mode of birth). Two sensitivity analyses were performed, complete case analysis and analysis where missing variables were imputed. Multiple imputation using chained equation was used to impute missing delivery related data that were essential to compute outcome variables. Missing values were imputed 28 times (since gestational age was missing among 28% of the women in the study) using linear (for gestational age and birth-weight outcomes), logistic (for stillbirth outcome and sex of the baby) and multinomial logistic (for delivery method categorical outcome) regression as appropriate. All analysis was performed in Stata version 17. Two-sided p value of < 0.05 was considered statistically significant.

Approval for this study was obtained from the Independent Scientific Advisory Committee for CPRD.

## Results

**Fig. 1:**
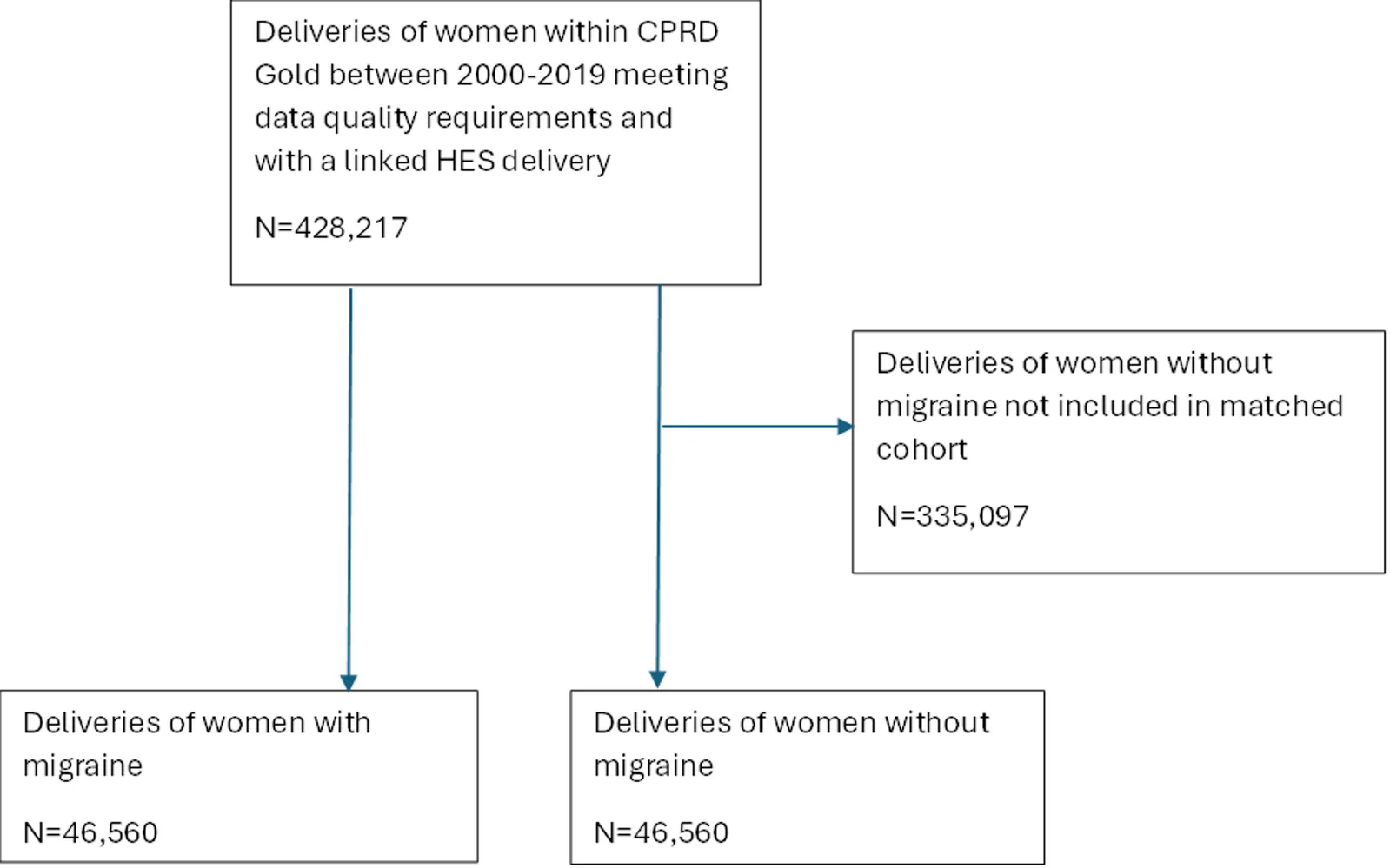
Study flow diagram. Flow chart to illustrate how study population for matched cohort was derived from deliveries of women within CPRD GOLD between 2000 and 2019 with linked HES delivery

There were 428,217 deliveries amongst 317,016 women meeting data quality criteria with a linked delivery in the HES maternity tail. Of these, 46,560 (10.9%) deliveries were from women with a history of migraine prior to pregnancy (Fig. 1). This was 1:1 matched to 46,560 deliveries from women without migraine.

### Baseline characteristics

The distribution of age and IMD were similar in the deliveries of women with and without migraine. However, a higher proportion of the women with migraine were White and fewer were from Black, South Asian, and other ethnic groups. The prevalence of obesity, smoking, pre-existing diabetes, gestational diabetes and pre-eclampsia were also higher in pregnancies of women with migraine (Table 1).

**Table 1:** Baseline characteristics of deliveries of women with migraine and matched cohort of women without migraine

	Deliveries of women with migraine	Deliveries of women without migraine
Number of deliveries	46,560	46,560
Number of women	34,506	42,851
Maternal Age, median (IQR)	29.9 (25.7–34.0)	30.0 (25.9–33.9)
Ethnicity, n (%)		
White	42,199 (90.6)	39,233 (84.3)
South Asian	1,553 (3.3)	2,349 (5.1)
Black	878 (1.9)	1,457 (3.1)
Mixed Ethnicity	437 (0.9)	536 (1.2)
Others	910 (2.0)	1,863 (4.0)
Missing	583 (1.3)	1,122 (2.4)
IMD, n (%)		
1 (Most deprived)	8,502 (18.3)	8,656 (18.6)
2	8,727 (18.7)	8,679 (18.6)
3	9,160 (19.7)	9,125 (19.6)
4	9,021 (19.4)	8,650 (18.6)
5 (Least deprived)	10,286 (22.1)	9,415 (20.2)
Missing	864 (1.9)	2,035 (4.4)
BMI (kg/m^2^), n (%)		
Underweight (< 18.5)	1,561 (3.4)	1,726 (3.7)
Normal weight (18.5-<25)	20,246 (43.5)	21,325 (45.8)
Overweight (25-<30)	10,224 (22.0)	9,716 (20.9)
Obese (≥ 30)	8,158 (17.5)	6,393 (13.7)
Missing	6,371 (13.7)	7,400 (15.9)
Smoking status, n (%)		
Non-smoker	24,470 (52.6)	25,968 (55.8)
Ex-smoker	7,681 (16.5)	6,863 (14.7)
Smoker	12,818 (27.5)	11,284 (24.2)
Missing	1,591 (3.4)	2,445 (5.3)
Maternal Morbidities, n (%)		
Asthma	10,944 (23.5)	7,098 (15.2)
Chronic kidney disease	20 (0.04)	22 (0.05)
Gestational diabetes	1,284 (2.8)	1,181 (2.5)
Hypertension	383 (0.82)	236 (0.51)
Thyroid disorder	646 (1.4)	551 (1.2)
Pre-existing diabetes	188 (0.40)	150 (0.32)
Systemic lupus erythematous	31 (0.07)	14 (0.03)

n=number of deliveries, BMI= Body Mass Index, IMD= Index of Multiple Deprivation

### Obstetric outcomes

#### Preterm birth

Among all deliveries, 8.32% (3,874) of women with migraine had preterm births, compared to 7.68% (3,574) of those without migraine. In deliveries where gestational age was recorded, 1.03% (481) of women with migraine had very preterm births and 0.88% (409) had extremely preterm births, compared to 1.27% (591) and 0.78% (363) in those without migraine, respectively (Table 2).

A small significant increase in the odds of preterm birth was found in the primary analysis treating missingness of gestational age as absence of preterm birth (Table 2) but this did not remain significant in the adjusted analysis (aOR 1.04 (0.99–1.09)). Similar results were found in the complete case analysis (Table 3). Where missing values were imputed, a small significant increase in the odds of preterm birth in women with migraine was found (aOR 1.06 (1.01–1.11)) (Table 4). Migraine was associated with decreased odds of very preterm birth in the primary analysis (aOR 0.83 (0.73–0.95)) (Table 2), which was replicated in the complete case analysis and the analysis of imputed values (Tables 3 and 4). There was no significant association between migraine and extremely preterm birth in the primary analysis (aOR 1.17 (1.00-1.36)) (Table 2). Where missing values were imputed, migraine was associated with a small, significant increased risk of extremely preterm birth (aOR 1.18 (1.01–1.37)) (Table 4) of the deliveries of women with migraine that were preterm, 2,344 (49.20%) were recorded as iatrogenic, and 2,216 (46.52%) were spontaneous, compared to 2,005 (44.28%) and 2,328 (51.41%) of those without migraine. Migraine was associated with an increased odds of iatrogenic preterm birth (aOR 1.10 (95% CI 1.04–1.18)), but not spontaneous preterm birth (aOR 0.97 (95%CI 0.91–1.03)) (Table 2).

#### Low birth weight

1,877 (4.03%) babies of women with migraine had low birth weight compared to 1,807 (3.88%) babies of those without migraine. 195 (0.42%) and 215 (0.46%) babies of women with migraine had very low birth weight and extremely low birth weight, respectively, compared to 191 (0. 41%) and 203 (0.44%) babies of women without migraine, respectively (Table 2). No significant association was found between migraine and low birth weight (aOR 1.02 (0.95–1.09)), very low birth weight (aOR 0.96 (0.78–1.19)) or extremely low birth weight (aOR 1.01 (0.83–1.22)) (Table 2). Similar results were found in the complete case analysis and analysis of imputed values. (Tables 3 and 4).

#### Small for gestational age

1,689 (3.63%) babies of women with migraine were small for gestational age compared to 1,771 (3.80%) babies of women without migraine. No significant association was found between migraine and small for gestational in the primary analysis treating missing as gestational age > 10th centile: aOR 0.96 (95% CI 0.89–1.03) (Table 2). This was replicated in the complete case analysis and analysis of imputed outcomes.

#### Pre-eclampsia

1,846 (3.96%) pregnancies of women with migraine were affected by pre-eclampsia compared to 1,596 (3.43% ) pregnancies of women without. Migraine was associated with a significant increase in the odds of pre-eclampsia (aOR 1.17 (95% CI 1.07–1.27)) (Table 2).

### Mode of delivery

36,764 (76.82%)of the deliveries of women with migraine were vaginal compared to 36,277 (77.92%) of those without. Of the deliveries in women with migraine, 5,097 (10.95%) and 5,699 (12.24%) were elective Caesarean and emergency Caesarean, respectively compared to 4,704 (10.10%) and 5,579 (11.98%) of deliveries of women without migraine. In the primary analysis, migraine was associated with an increased risk of elective caesarean delivery, compared to vaginal delivery but this did not remain significant after adjustment (aOR 1.04 (95% CI 0.99–1.09)). There were similar findings in the complete case analysis and analysis of imputed outcomes. No significant association was found between migraine and risk of emergency section in any analysis (primary analysis: aOR 1.01 (95% CI 0.97–1.06). (Tables 2, 3 and 4).

Stillbirth182 (0.39%) of babies of women with migraine were stillborn compared to 177 (0.38%) of babies of women without migraine. No significant association was found between migraine and stillbirth (aOR 1.00 (95% CI 0.81–1.24) (Table 2).

**Table 2:** Obstetric outcomes for deliveries of women with and without migraine (treating missing as absence of outcome (or missing delivery as vaginal delivery))

	Deliveries of women with migraine, *n* = 46.560	Deliveries of women without migraine, *n* = 46,560	OR	aOR*
Gestational age at delivery, *n* (%)				
Term (≥ 37 weeks)	41,796 (89.77)	42,032 (90.27)	Ref	Ref
	*including 12,326 (26.47%) gestational age not recorded	* including 12,660 (27.19%) not recorded		
Preterm birth (32–37 weeks gestational age or coded as preterm in record)	3,874 (8.32)	3,574 (7.68)	1.09 (1.04–1.14)	1.04 (0.99–1.09)
Very preterm (28–32 weeks)	481 (1.03)	591 (1.27)	0.82 (0.72–0.93)	0.83 (0.73–0.95)
Extremely preterm (< 28 weeks)	409 (0.88)	363 (0.78)	1.13 (0.97–1.32)	1.17 (1.00-1.36)
Onset of preterm birth, n (%)				
Spontaneous preterm birth	2,216 (46.52)	2,328 (51.41)	0.96 (0.90–1.02)	0.97 (0.91–1.03)
Iatrogenic preterm birth	2,344 (49.20)	2,005 (44.28)	1.18 (1.10–1.25)	1.10 (1.04–1.18)
Onset of delivery not recorded	204 (4.28)	195 (4.31)	1.05 (0.86–1.28)	1.04 (0.85–1.27)
Birthweight, n (%)				
Birthweight ≥ 2500 g	44,273 (95.09)	44,359 (95.27)	Ref	Ref
	*Including 7,953 (17.08) birthweight not recorded	*Including 8,317 (17.86) birthweight not recorded		
LBW (1500–2500 g)	1,877 (4.03)	1,807 (3.88)	1.04 (0.97–1.11)	1.02 (0.95–1.09)
VLBW (1000–1500 g)	195 (0.42)	191 (0.41)	1.02 (0.84–1.25)	0.96 (0.78–1.19)
ELBW (< 1000 g)	215 (0.46)	203 (0.44)	1.06 (0.87–1.29)	1.01 (0.83–1.22)
Birthweight for gestational age, n (%)				
Gestational age > 10th percentile	30,722 (65.98)	30,387 (65.26)	Ref	Ref
Small for gestational age (10th percentile or below)	1,689 (3.63)	1,771 (3.80)	0.94 (0.88–1.01)	0.96 (0.89–1.03)
Missing	14,149 (30.39)	14,402 (30.93)		
Pre-eclampsia, n (%)	1,846 (3.96)	1,596 (3.43)	1.16 (1.09–1.25)	1.17 (1.07–1.27)
Mode of delivery, n (%)				
Vaginal delivery	36,764 (76.82)	36,277 (77.92)	Ref	Ref
	*Including 6,829 (14.67) mode not recorded	*Including 7,318 (15.72) mode not recorded		
Elective caesarean section	5,097 (10.95)	4,704 (10.10)	1.08 (1.03–1.14)	1.04 (0.99–1.09)
Emergency caesarean section	5,699 (12.24)	5,579 (11.98)	1.02 (0.98–1.07)	1.01 (0.97–1.06)
Stillbirth, n (%)	182 (0.39)	177 (0.38)	1.03 (0.84–1.27)	1.00 (0.81–1.24)

N= number of deliveries, LBW = Low Birth Weight, VLBW= Very Low Birth Weight, ELBW= Extremely Low Birth Weight

**Table 3:** Odds ratios and 95% confidence intervals for mode of delivery for deliveries of women with migraine compared to those without (complete case analysis)

			Matched Cohort	
			OR	aOR*
Gestational age at delivery				
Preterm Birth (32–37 weeks)	3,874 (8.32)	3,574 (7.68)	1.08 (1.03–1.14)	1.04 (0.99–1.10)
Very preterm (28–32 weeks)	481 (1.03)	591 (1.27)	0.81 (0.71–0.93)	0.83 (0.73–0.95)
Extremely preterm (< 28 weeks)	409 (0.88)	363 (0.78)	1.13 (0.97–1.31)	1.17 (1.00-1.37)
Birthweight				
LBW (15002500 g)	2,287 (4.91)	2,201 (4.73)	1.03 (0.96–1.10)	1.02(0.95–1.09)
VLBW (10001500 g)	410 (0.88)	394 (0.85)	1.01 (0.83–1.23)	0.97(0.79–1.19)
ELBW (< 1000 g)	215 (0.46)	203 (0.44)	1.05 (0.87–1.28)	1.0(0.82–1.22)
Birthweight for gestational age				
Small for gestational age (< 10th centile)	1,689 (3.63)	1,771 (3.80)	0.94 (0.88–1.01)	0.96 (0.89–1.03)
Mode of delivery				
Elective caesarean section	5,097 (10.95)	4,704 (10.10)	1.08 (1.03–1.14)	1.04 (0.99–1.09)
Emergency caesarean section	5,699 (12.24)	5,579 (11.98)	1.02 (0.98–1.07)	1.01 (0.97–1.06)

* Adjusted for age, ethnicity, body mass index, smoking status, year of pregnancy, delivery method, pre-eclampsia, gestational diabetes, history of diabetes, asthma, systemic lupus erythematous, thyroid disorders, hypertension, chronic kidney diseaseLBW = Low Birth Weight, VLBW= Very Low Birth Weight, ELBW= Extremely Low Birth Weight

**Table 4:** Odds ratios and 95% confidence intervals for mode of delivery for deliveries of women with migraine compared to those without (missing outcomes imputed)

			Matched Cohort	
			OR	aOR*
Gestational age at delivery				
Term (≥ 37 weeks)	41,320 (88.75)	41,631 (89.4)	ref	ref
Preterm Birth (32–37 weeks)	4,275 (9.18)	3,915 (8.41))	1.10 (1.05–1.15)	1.06 (1.01–1.11)
Very preterm (28–32 weeks)	546 (1.17)	642 (1.38)	0.86 (0.76–0.97)	0.87 (0.77–0.99)
Extremely preterm (< 28 weeks)	419 (0.90)	372 (0.8)	1.13 (0.98–1.32)	1.18 (1.01–1.37)
Birthweight				
Birthweight ≥ 2500 g	44,217 (94.97)	44,341 (95.23)	ref	ref
LBW (1500–2500 g)	1,931 (4.15)	1,825 (3.92)	1.06 (0.99–1.13)	1.06 (0.99–1.14)
VLBW (1000–1500 g)	197 (0.42)	191 (0.41)	1.03 (0.85–1.26)	1.00 (0.82–1.23)
ELBW (< 1000 g)	215 (0.46)	203 (0.44)	1.06 (0.87–1.29)	1.02 (0.84–1.24)
Birthweight for gestational age				
Gestational age > 10th percentile	44,798 (96.2)	44,698 (96)	ref	ref
Small for gestational age (< 10th centile)	1,762 (3.78)	1,862 (4)	0.94 (0.88–1.01)	0.96 (0.89–1.03)
Mode of delivery				
Vaginal Delivery	34,016 (73.1)	34,393 (73.9)		
Elective caesarean section	5,937 (12.8)	5,562 (12.0)	1.08 (1.03–1.13)	1.06 (0.97–1.16)
Emergency caesarean section	6,607 (14.2)	6.605 (14.2)	1.01 (0.98–1.05)	1.00 (0.92–1.09)

* Adjusted for age, ethnicity, body mass index, smoking status, year of pregnancy, delivery method, pre-eclampsia, gestational diabetes, history of diabetes, asthma, systemic lupus erythematous, thyroid disorders, hypertension, chronic kidney diseaseLBW = Low Birth Weight, VLBW= Very Low Birth Weight, ELBW= Extremely Low, Birth Weight

## Discussion

### Principal findings

This retrospective cohort study used primary care routine health records linked to hospital maternity data in England to investigate the relationship between migraine and preterm birth, low birth weight, small for gestational age, pre-eclampsia, mode of delivery and stillbirth. Maternal migraine was associated with an increased odds of iatrogenic preterm delivery but appeared to have a protective effect against very preterm delivery. In a sensitivity analysis where missing outcomes were imputed, pre-pregnancy migraine was associated with a small but significant increase in the odds of preterm and extremely preterm delivery. Maternal migraine was also found to be associated with an increased odds of pre-eclampsia. No significant associations were found between migraine and the remaining obstetric outcomes.

### Strengths and Limitations

This study has several strengths, including over 40,000 births from a UK-representative database, making it one of the largest studies on obstetric outcomes in women with migraine. With over 97% of births occurring in NHS hospitals [34] the sample represents UK population. Using linked primary care data allowed inclusion of more migraine cases than previous studies relying on secondary care diagnosis [22, 23]. We distinguished between spontaneous and iatrogenic preterm delivery, shedding light on mechanisms behind the association with migraine. This is the first study to separate elective and emergency Caesarean sections in the migraine population, considering differing risks and indications [26, 27]. We adjusted for important factors like maternal age, smoking, and conditions during pregnancy, and restricted the analysis to singleton deliveries to reduce confounding [35].

However, the migraine prevalence in this study was 10.9%, lower than the ~ 20% in other studies (1), which may indicate unrecorded cases in the control group (3). Despite excluding women with other headache disorders, some may have remained undiagnosed. Additionally, Black, South Asian, and other ethnic groups were under-represented in the migraine cohort, potentially reflecting both true prevalence differences and barriers to care [36, 37]. We were unable to examine the impact of migraine subtypes or severity on the outcomes. Further to this, we were also unable to examine the duration that migraine had been experienced prior to pregnancy or the frequency of migraine prior to or during pregnancy. As patients may self-manage migraine for some time before consulting with their GP and are unlikely to report every episode of migraine, these are likely to not be well-recorded within primary care records. Migraine with aura has been found to be associated with a higher vascular risk [38]. However, aura status was only present in the structured data in a small proportion of women. Although we could determine if preterm delivery was medically indicated, the specific reason, whether maternal or fetal, was not available. Small for gestational age is often used as a proxy for fetal growth restriction, or failure of the fetus to reach its growth potential [39]. While small for gestational age (SGA) is often used as a proxy for fetal growth restriction, it does not capture growth deceleration throughout pregnancy [40, 41]. Missing data, ranging from 14 to 30%, was addressed by sensitivity analysis, showing little impact on results. Unmeasured confounders, such as congenital anomalies, infections, and anaemia [42] may have influenced findings, as may the risk of congenital anomalies linked to migraine [43]. For some women, more than one pregnancy was included in the cohort. Whilst an adjustment was made for this, having migraine in a previous pregnancy may have affected the baseline risk of outcomes in a subsequent pregnancy. The study did not examine the effect of migraine medications on the outcomes studied. Finally, multiple outcomes were studied, increasing the likelihood of significant results by chance [44].

### Results in the context of what is known

The lack of a significant association between migraine and preterm birth in our primary analysis contrasts with two large database analyses. A Danish birth cohort found migraine linked to a higher risk of preterm (adjusted Prevalence Ratio (aPR) 1.21 [1.13–1.30]) and very preterm birth (aPR 1.35 [1.14–1.60]) (22) while a Taiwanese study also found a higher odds of preterm birth (aOR 1.24 [1.13–1.39]) [23]. Both studies relied on secondary care-recorded migraine diagnoses, likely reflecting more severe cases. It is possible that more severe migraine is associated with preterm birth, which may explain the absence of a higher overall risk in our study.

A US study of nurses, relying on self-reported (physician-diagnosed) exposures and outcomes, found a significantly higher risk of preterm birth in women with migraine (aRR 1.17 [1.01–1.35] without aura, aRR 1.17 [1.01–1.36] with aura) [24] with a similar prevalence to ours (11.3%). Differences in practices between countries and over time may explain this. The study enrolled women from 1989 to 2009 and surveyed them about previous pregnancies, which may have occurred earlier. Preterm birth increased in the US during the 1990–2000 s, linked to more obstetric interventions, followed by efforts to reduce elective deliveries before 39 weeks [45, 46]. However, as none of these studies differentiated between spontaneous or indicated preterm birth, it is unclear if this accounts for the differing findings.

Small studies differentiating between spontaneous and iatrogenic preterm birth have shown inconsistent results. A US study found no significant association between migraine and either spontaneous (aOR 0.79 [0.51–1.26]) or iatrogenic (aOR 1.03 [0.57–1.85]) preterm birth, but it included only 550 women with migraine [47]. Conversely, another US study found migraine linked to a higher odds of both iatrogenic (aOR 1.44 [1.08–1.89]) and spontaneous preterm birth (aOR 1.40 [1.11–1.77]) [33] with a higher prevalence of migraine (19.1%), suggesting more complete capture. Our study may have included women with unrecorded migraine in the unexposed group, potentially diluting the association with preterm birth.

In contrast to this study, two previous studies reported a significant increase in the risk of low birth weight. Skajaa et al. found a 14% increase (aPR 1.14 [1.06–1.23]) [22] and Chen et al. found a 16% increase (aOR 1.16 [1.03–1.31]) [23]. No study has found an association between migraine and small for gestational age, though a meta-analysis reported a small significant association (pooled aOR 1.08 [1.01–1.15]) [12].

The findings of a significant association between migraine and pre-eclampsia are in keeping with those found in a systematic review and meta-analysis, which reported a pooled adjusted odds ratio of 2.22 (95%CI 1.34–3.68) [12].

The lack of an association between migraine and caesarean delivery in this study contrasts with Danish and Taiwanese cohort studies, which found an aPR 1.20 [1.15–1.25] and aOR 1.16 [1.07–1.24] respectively [22, 23]. This may reflect differences in migraine severity across populations. Finally, the finding of no significant association between migraine and stillbirth aligns with other studies [22, 33].

### Clinical implications

The association between migraine and iatrogenic preterm birth, but not spontaneous preterm birth, suggests other underlying risk factors, such as the established link between migraine and pre-eclampsia [12], which often leads to preterm delivery [48]. The association persisted after adjusting for pre-eclampsia, but confounding factors may remain. Other conditions commonly comorbid with migraine, such as asthma, hypertension, CKD, hyperthyroidism, and SLE, were also adjusted for [21]. These factors may explain the association between migraine and elective caesarean delivery, as no significant link remained after adjustment. Unadjusted factors like placental complications [49], including a potential association with migraine, and fetal indications for preterm birth, such as growth restriction [50, 51] should also be considered. The apparent protective effect of migraine against very preterm birth has not been previously reported and mechanisms are unclear and warrant further investigation.

### Research implications

The underlying medical indications for preterm delivery in women with migraine need further exploration, particularly the role of pre-eclampsia and comorbid long-term conditions. Although no association was found between migraine and SGA, issues with the SGA definition in this study must be considered. Fetal indications for preterm delivery in women with migraine should also be examined. The association between migraine and pre-eclampsia, potentially linked by common mechanisms such as pro-inflammatory states and endothelial dysfunction, suggests that women with migraine may have compromised vascular compensatory mechanisms during pregnancy, increasing the likelihood of pre-eclampsia [52]. Pre-eclampsia is linked to placental dysfunction, which impairs uteroplacental blood flow and is a major cause of intrauterine growth restriction [53, 54]. Previous studies have also shown a link between migraine and SGA [12]. Future research could use more reliable measures like customised growth charts or track growth deceleration throughout pregnancy [33] to better assess this association [40, 55].

Further investigation is needed into how the severity and type of migraine influence obstetric outcomes. Variations in migraine severity across studies may explain the differences in findings, as severity likely affects treatment choices during pregnancy, influencing outcomes. Related to this, the effects of migraine medications on obstetric outcomes also warrants further investigation. Since migraine with aura is associated with higher vascular risk [38] examining the impact of migraine type on obstetric outcomes may also be beneficial. Limitations in routinely collected healthcare data, such as poorly recorded aura status and migraine severity, underscore the need for prospective studies that track women from pre-conception through pregnancy.

## Conclusions

In this large delivery cohort study, migraine was associated with an increased risk of pre-eclampsia and iatrogenic preterm delivery. Further work is required to understand the potential reasons behind these associations.

## Supplementary Information

Below is the link to the electronic supplementary material.

Supplementary Material 1

## Data Availability

The data that support the findings of this study are available from CPRD but restrictions apply to the availability of these data, which were used under license for the current study, and so are not publicly available.The Read codes and International Classification of Disease-version 10 (ICD-10) codes used in this paper are available from: [https://github.com/mumpredict/Read-codes-and-ICD-10-codes](https:/github.com/mumpredict/Read-codes-and-ICD-10-codes).

## Abbreviations

CPRD GOLD: Clinical Practice Research Datalink GOLD
HES: Hospital Episodes Statistics
SGA: Small for Gestational Age
NSAIDs: Non-steroidal anti-inflammatory drugs
EHR: Electronic health record
GP: General practitioner
NHS: National Health Service
CGRP: Calcitonin gene-related peptide
ICD: International Classification of Disease
LBW: Low Birth Weight
VLBW: Very Low Birth Weight
ELBW: Extremely Low Birth Weight
OPCS: Office of Population Censuses and Surveys Classification of Interventions and Procedures
DExtER: Data Extraction for Epidemiology Research
IMD: Index of Multiple Deprivation
BMI: Body Mass Index
